# Tidal lung hysteresis to interpret PEEP-induced changes in compliance in ARDS patients

**DOI:** 10.1186/s13054-023-04506-6

**Published:** 2023-06-13

**Authors:** Francesco Mojoli, Marco Pozzi, Eric Arisi, Silvia Mongodi, Anita Orlando, Giuseppe Maggio, Federico Capra Marzani, Laurent Brochard

**Affiliations:** 1 Department of Clinical-Surgical, Diagnostic and Pediatric Sciences, Unit of Anesthesia, University of Pavia, Pavia, Italy; 2 Anesthesia and Intensive Care, Fondazione IRCCS Policlinico S. Matteo, Pavia, Italy; 3 Keenan Centre for Biomedical Research, Li Ka Shing Knowledge Institute, Unity Health Toronto, Toronto, ON Canada; 4 Interdepartmental Division of Critical Care Medicine, University of Toronto, Toronto, ON Canada

**Keywords:** PEEP setting, Decremental PEEP trial, Tidal recruitment, Lung hysteresis, Lung recruitability, Pressure–volume curves

## Abstract

**Background:**

In ARDS, the PEEP level associated with the best respiratory system compliance is often selected; however, intra-tidal recruitment can increase compliance, falsely suggesting improvement in baseline mechanics. Tidal lung hysteresis increases with intra-tidal recruitment and can help interpreting changes in compliance. This study aims to assess tidal recruitment in ARDS patients and to test a combined approach, based on tidal hysteresis and compliance, to interpret decremental PEEP trials.

**Methods:**

A decremental PEEP trial was performed in 38 COVID-19 moderate to severe ARDS patients**.** At each step, we performed a low-flow inflation-deflation manoeuvre between PEEP and a constant plateau pressure, to measure tidal hysteresis and compliance.

**Results:**

According to changes of tidal hysteresis, three typical patterns were observed: 10 (26%) patients showed consistently high tidal-recruitment, 12 (32%) consistently low tidal-recruitment and 16 (42%) displayed a biphasic pattern moving from low to high tidal-recruitment below a certain PEEP. Compliance increased after 82% of PEEP step decreases and this was associated to a large increase of tidal hysteresis in 44% of cases. Agreement between best compliance and combined approaches was accordingly poor (K = 0.024). The combined approach suggested to increase PEEP in high tidal-recruiters, mainly to keep PEEP constant in biphasic pattern and to decrease PEEP in low tidal-recruiters. PEEP based on the combined approach was associated with lower tidal hysteresis (92.7 ± 20.9 vs. 204.7 ± 110.0 mL; *p* < 0.001) and lower dissipated energy per breath (0.1 ± 0.1 vs. 0.4 ± 0.2 J; *p* < 0.001) compared to the best compliance approach. Tidal hysteresis ≥ 100 mL was highly predictive of tidal recruitment at next PEEP step reduction (AUC 0.97; *p* < 0.001).

**Conclusions:**

Assessment of tidal hysteresis improves the interpretation of decremental PEEP trials and may help limiting tidal recruitment and energy dissipated into the respiratory system during mechanical ventilation of ARDS patients.

**Supplementary Information:**

The online version contains supplementary material available at 10.1186/s13054-023-04506-6.

## Background

ARDS is a heterogeneous syndrome with variable response to specific treatments [1–3]. Some evidence suggests that PEEP setting during mechanical ventilation should be tailored to optimize patient’s respiratory mechanics [4–6]. The “best compliance” approach is based on the changes of respiratory system compliance observed during a decremental PEEP trial [7]. An increase in compliance when PEEP is reduced is interpreted as a substantial decrease of the number of hyper-inflated alveoli; at the opposite, as soon as the compliance starts to decrease, this is interpreted as substantial lung derecruitment. However, PEEP changes can also promote cyclic opening and closing of alveoli and distal airways [5, 8, 9]. Intra-tidal recruitment represents a considerable dissipation of energy within the lung [5, 8, 9] and is associated with an increase of the measured compliance of the respiratory system during the insufflation [9–11]. Thus, tidal recruitment complicates the interpretation of a decremental PEEP trial based on compliance: in fact, a “better” compliance after a PEEP step-down can be due to a favourable mechanical effect (lower number of overdistended alveoli) or alternatively to an unfavourable mechanical effect (greater amount of tidal recruitment). This non univocal interpretation of compliance changes may help explain why the best compliance approach did not improve the outcome of ARDS patients [12].

At the bedside, lung recruitability can be assessed with static pressure–volume loops obtained during low-flow inflation-deflation manoeuvres: the area of hysteresis (i.e., the area enclosed between inflation and deflation limbs of the curve) is correlated with lung recruitment [13–15]. Modern ventilators allow to obtain low-flow pressure–volume loops starting from different PEEP values and exploring the range of pressures corresponding to tidal ventilation, from PEEP to plateau pressure (i.e., tidal pressure–volume loops). Tidal lung hysteresis can then be measured and used to assess tidal recruitment associated with a specific PEEP value [13]. This information could help physicians to interpret changes of compliance during a decremental PEEP trial. The aims of this study are (1) to describe tidal lung hysteresis as a function of PEEP in ARDS patients and (2) to test the hypothesis that the evaluation of compliance and tidal hysteresis combined, compared to compliance alone, can substantially change the interpretation of a decremental PEEP trial in these patients.

## Materials and methods

We enrolled moderate to severe COVID-19 ARDS patients when the attending physician performed a decremental PEEP trial to personalize the ventilatory settings. This was considered as a second-line approach in our centre, adopted in difficult cases when our standard approach, based on lung morphology [3, 16, 17] and/or calibrated esophageal pressure [18, 19], was considered by the medical team not fully satisfactory and when the required equipment was available. The study was approved by the local ethics committee (N. 20210090319). Consent for data collection was obtained at hospital admission from all individual participants included in the study. Patients were sedated, paralyzed, connected to a G5 or C6 mechanical ventilator (Hamilton Medical AG, Bonaduz, Switzerland) equipped with a tool to perform a low-flow inflation/deflation manoeuvre (PV tool) and ventilated in pressure-controlled ventilation mode through a respiratory circuit with heated humidifier.

### Decremental PEEP trial execution

A recruitment manoeuvre was performed by increasing with a constant rate (5 cmH_2_O/s) the airway pressure from clinical PEEP to an upper value of 35–40 cmH_2_O, then maintained for 10 s. After the recruitment manoeuvre, PEEP was set at the clinical PEEP + 6 cmH_2_O (PEEP start) and decreased by 2 cmH_2_O every 3 min until clinical PEEP – 6 cmH_2_O (PEEP end)). The range of PEEP tested could be set differently by the attending physician in case of hemodynamic instability and/or desaturation. The driving pressure (ΔP) was set at PEEP start to keep plateau pressure close to 30 cmH_2_O. Plateau pressure was kept constant throughout the decremental PEEP trial, therefore the applied ΔP increased by 2 cmH_2_O at each step (Additional file 1: Fig S1). Doing so, we tested the ability of different PEEP values to keep open the lung tissue that was recruited by the same opening pressure. Respiratory rate was adjusted along the trial to have at end-inspiration and end-expiration a nearly zero flow condition (i.e., an alveolar pressure reflected by airway pressure) and to keep end-tidal CO_2_ reasonably constant. At the end of each PEEP step, a low-flow inflation/deflation manoeuvre was performed, from PEEP to plateau pressure and back; the inflation rate of increase/decrease of airway pressure was set at 2 cmH_2_O/s during the whole manoeuvre.

### Decremental PEEP trial analysis

**Fig. 1:**
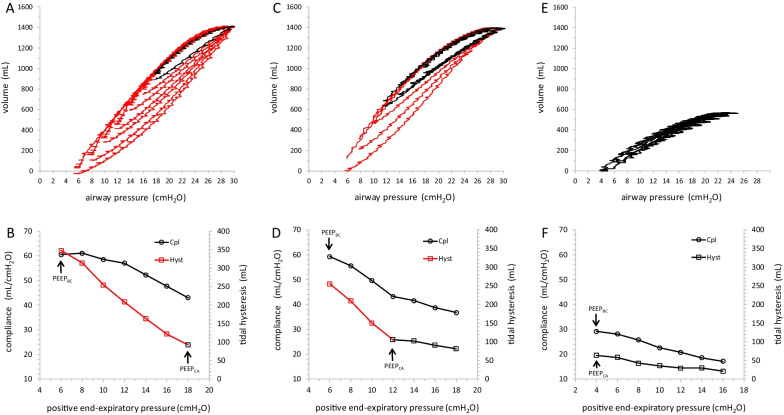
Decremental pressure–volume loops in COVID-19 ARDS patients with three different patterns of tidal lung recruitability. Pressure–volume loops obtained during low-flow inflation-deflation manoeuvers at decremental PEEP levels and constant plateau pressure in 3 patients with high, biphasic and low tidal recruitability are displayed in panels A, C and E respectively. At each PEEP step-down, the change of end-expiratory lung volume was computed as the difference between two consecutive expiratory volumes, before and after the PEEP change. All the curves are displayed on the same volume axis, where the value of zero corresponds to the end-expiratory lung volume at the lowest value of PEEP tested. The PV curve is red if associated to a large increase in tidal lung hysteresis compared to the previous PEEP step, otherwise is black. Corresponding values of respiratory system compliance (Cpl, open circles) and of tidal lung hysteresis (Hyst, open squares) are displayed in panels B, D and F. Values of Hyst are displayed as red open squares when associated to a large increase of its value compared to the previous PEEP step, otherwise are displayed as black open squares. PEEP values suggested by the best compliance approach (PEEP_BC_) and by the combined approach (PEEP_CA_) are also displayed. In the patient with high tidal recruitability (panels A and B) at each step down of PEEP, tidal hysteresis consistently showed a large increase suggesting progressive tidal recruitment moving from the highest to the lowest PEEP value. Respiratory system compliance increased rapidly moving from PEEP 18 to PEEP 10 and thereafter was almost constant; this can be explained with the effect of tidal recruitment alone (from PEEP 18 to 10) with increased estimate of compliance and with a combination of lung derecruitment and tidal recruitment (from PEEP 10 to PEEP 6) with opposite and counterbalancing effects on compliance. Best compliance PEEP (PEEP_BC_) was 6 cmH_2_O and combined approach PEEP (PEEP_CA_) was 18 cmH_2_O. In the patient with biphasic pattern (panels C and D), PV loops obtained from PEEP 18 to PEEP 12 were almost superimposed: this was associated with a small and progressive increase of both tidal hysteresis and compliance, suggesting that a PEEP of 12 cmH_2_O was still effective in avoiding tidal recruitment and lung derecruitment. Further decrease of PEEP (from 10 to 6 cmH_2_O) was associated with large increase of tidal hysteresis and compliance, suggesting that PEEP was not anymore able to prevent progressive tidal recruitment. PEEP_BC_ was 6 cmH_2_O and PEEP_CA_ was 12 cmH_2_O. In the patient with low tidal recruitability (panels E and F) at each PEEP step down both tidal hysteresis and compliance consistently showed a small and progressive increase, suggesting that also the lowest value of PEEP was still able to prevent tidal recruitment and lung derecruitment. Both PEEP_BC_ and PEEP_CA_ were 4 cmH_2_O

The pressure–volume curves generated by the low-flow inflation/deflation manoeuvres were recorded for off-line analysis. Standard correction for gas exchange was applied [15]. The compliance of the respiratory system was computed from the inflation limb of the curve as ΔVolume/ΔP and expressed in mL/cmH_2_O. At each PEEP step-down, the change in compliance between two PEEP levels (ΔCpl) was computed; compliance was considered increased or decreased when ΔCpl was of at least 1 ml/cmH_2_O in absolute value. The area of the hysteresis of the tidal pressure–volume loop was computed and its value was normalized to the actual ΔP: tidal lung hysteresis (Hyst) = Hysteresis area (mL * cmH_2_O) / ΔP (cmH_2_O); Hyst was therefore expressed in mL. After a reduction in PEEP, a large increase of tidal lung hysteresis was considered a marker of substantial increase of tidal recruitment [13, 20, 21]. The change of tidal lung hysteresis associated with each PEEP step-down was computed as ΔHyst/ΔPEEP in mL/cmH_2_O. A threshold of 10 mL/cmH_2_O was used to differentiate small from large change of tidal hysteresis based on the distribution of data (Additional file 2: Fig. S2). Patients displayed three patterns of tidal recruitability: “high”, when they consistently showed large increase of Hyst at each PEEP step-down, “low” when they showed consistently small increase of Hyst, and a biphasic response with small increase of Hyst at high PEEP values and large increase of Hyst below a certain PEEP value. Figure 1 shows pressure–volume loops, compliance and tidal lung hysteresis at decremental PEEP levels in three representative patients with high, biphasic and low tidal recruitability. To assess the amount of energy dissipated into the respiratory system per breath, the area of hysteresis of the tidal pressure–volume loop was expressed in Joules: dissipated Energy (J) = 0.098 * Hysteresis area (mL*cmH_2_O) / 1000.

### Decremental PEEP trial interpretation: best compliance Vs. combined approach

According to the best compliance approach, the mechanical effect of a PEEP step-down was considered unfavourable when the compliance decreased. The PEEP level chosen by the “best compliance” approach (PEEP_BC_) was the last PEEP value before a decrease in compliance was observed. In the combined approach, compliance and lung hysteresis were both considered: the mechanical effect of a PEEP step-down was considered unfavourable in case of a decrease of compliance and/or a large increase of Hyst. The PEEP level suggested by the combined approach (PEEP_CA_) was the last one before any unfavourable mechanical effect.

### Statistical analysis

The interrater agreement between the two approaches was assessed with Cohen’s K coefficient in terms of suggested PEEP change (increase/keep constant/decrease from clinical PEEP). Considering a value of K1 = 0.3 as the upper limit for a random agreement and a value of K2 = 0.7 as the lowest limit for acceptable agreement, a sample size of 34 patients was required to achieve a power of 90% with an alpha error of 0.05 [22]. The two approaches were also compared in terms of suggested PEEP with paired T-test. Tidal lung hysteresis, dissipated energy and compliance were compared among clinical PEEP, PEEP_BC_ and PEEP_CA_ with repeated measures analysis of variance (ANOVA). One-way ANOVA was used to compare patients with different recruitability patterns. ROC curves’ analysis was performed to test sensitivity/specificity of tidal lung hysteresis to predict a large increase of tidal recruitment at next PEEP step-down. No missing data were expected.

## Results

We analysed 38 patients with moderate to severe COVID-19 ARDS, admitted to our ICU between March 2020 and June 2021. Patients were male 30/38 (78.9%) and 60.1 ± 9.2 year-old; at the time of the trial, their median hospital stay was 7.5 (IQR 4.2–15.2) days and the median length of invasive mechanical ventilation was 4.8 (IQR 1.8–12.3) days. PaO_2_/FiO_2_ ratio was lower than 150 mmHg in 31/38 (81.6%) and 7/38 (18.4%) were on veno-venous extra-corporeal membrane oxygenation (ECMO). Ventilator settings, respiratory mechanics and gas exchanges recorded right before the trial are detailed in Table 1. During the trial, PEEP was decreased by steps from 20.4 ± 2.8 to 9.6 ± 3.0 cmH_2_O, while keeping plateau pressure at 30.5 ± 2.3 cmH_2_O (Table 2).

**Table 1:** Demographics, ventilator settings, respiratory mechanics and gas exchanges in all patients and in subgroups with different mechanical patterns

	All patients *N* = 38 (100.0%)	High tidal-recruiters *N* = 10 (26.3%)	Biphasic tidal-recruiters *N* = 16 (42.1%)	Low tidal-recruiters *N* = 12 (31.6%)	*P* value
Age (years)	60.1 ± 9.2	63.1 ± 7.4	60.3 ± 8.7	57.4 ± 11.1	0.364
Male gender (*n*, %)	30 (78.9)	8 (80.0)	13 (81.3)	9 (75.0)	1.000
Hospital stay (days)	7.5 (4.2–15.2)	4.7 (1.8–5.8)	10.2 (3.4–13.1)	17.2 (6.6–26.1)	0.005
MV length (days)	4.8 (1.8–12.3)	1.8 (0.9–4.8)	5.8 (1.1–9.7)	14.5 (2.6–24.0)	0.014
ECMO (n, %)	7 (18.4)	0 (0.0)	5 (31.3)	2 (16.7)	0.158
PEEP (cmH_2_O)	15.3 ± 2.5	16.3 ± 2.8	15.3 ± 2.5	14.4 ± 2.2	0.220
*P*-plateau (cmH_2_O)	28.0 ± 2.3	28.7 ± 2.2	28.2 ± 2.4	27.2 ± 2.1	0.331
ΔP (cmH_2_O)	12.7 ± 0.6	12.4 ± 1.1	12.9 ± 0.3	12.8 ± 0.4	0.092
Cpl (mL/cmH_2_O)	33.9 ± 12.9	46.4 ± 11.1	36.1 ± 7.6	20.5 ± 6.2	< 0.001
TV (mL)	427.0 ± 148.2	565.2 ± 96.7	464.5 ± 91.2	261.9 ± 75.9	< 0.001
FiO_2_	0.7 ± 0.2	0.7 ± 0.2	0.7 ± 0.1	0.6 ± 0.2	0.272
PaO_2_ (mmHg)	80.9 ± 17.0	82.8 ± 20.0	77.9 ± 19.3	82.3 ± 12.3	0.764
PaCO_2_ (mmHg)	55.8 ± 10.5	52.2 ± 7.3	54.7 ± 11.7	59.8 ± 10.8	0.221

Data are related to the time at which the decremental PEEP trial was performed in each single patient. Clinical setting of mechanical ventilation is displayed. *MV* Mechanical ventilation, *ECMO* Extracorporeal membrane oxygenation, *PEEP* Positive end-expiratory pressure, *P*-plateau Plateau pressure, Δ*P* Driving pressure, *Cpl* Respiratory system compliance, *TV* Tidal volume, FiO_2_ Inspiratory fraction of oxygen, PaO_2_ Arterial partial pressure of oxygen, PaCO_2_ Arterial partial pressure of carbon dioxide

**Table 2:** Decremental PV loops: ventilator settings and respiratory mechanics findings in all patients and in subgroups with different mechanical patterns

	All patients *N* = 38 (100.0%)	High tidal-recruiters *N* = 10 (26.3%)	Biphasic tidal-recruiters *N* = 16 (42.1%)	Low tidal-recruiters *N* = 12 (31.6%)	*P* value
PEEP start (cmH_2_O)	20.4 ± 2.8	19.5 ± 3.1	21.4 ± 2.3	19.9 ± 3.0	0.085
PEEP end (cmH_2_O)	9.6 ± 3.0	10.1 ± 3.7	9.7 ± 2.9	8.9 ± 2.7	0.696
ΔPEEP (cmH_2_O)	10.9 ± 2.4	9.4 ± 3.0	11.8 ± 2.2	11.0 ± 1.8	0.047
P-Plateau (cmH_2_O)	30.5 ± 2.3	30.4 ± 1.6	30.6 ± 2.0	30.2 ± 3.2	0.789
Cpl start (mL/cmH_2_O)	29.6 ± 11.3	42.8 ± 7.3	30.2 ± 5.9	17.7 ± 4.6	< 0.001
Cpl end (mL/cmH_2_O)	44.7 ± 17.2	58.4 ± 16.3	49.0 ± 11.8	27.5 ± 8.5	< 0.001
Hyst start (mL)	63.8 ± 29.3	100.6 ± 20.0	62.0 ± 14.9	35.7 ± 12.5	< 0.001
Hyst end (mL)	212.2 ± 111.1	335.4 ± 82.8	230.5 ± 49.0	85.3 ± 27.0	< 0.001
ΔHyst/ΔPEEP (mL/cmH_2_O)	14.4 ± 9.9	25.9 ± 10.0	14.5 ± 4.0	4.5 ± 1.6	< 0.001
Dissipated E start (J)	0.1 ± 0.0	0.1 ± 0.0	0.1 ± 0.0	0.0 ± 0.0	< 0.001
Dissipated E end (J)	0.4 ± 0.2	0.7 ± 0.2	0.5 ± 0.1	0.1 ± 0.1	< 0.001

Data are provided for the higher (start values) and the lower (end values) level of PEEP tested in the decremental PEEP trial. *PEEP* Positive end-expiratory pressure, Δ*PEEP* Difference between PEEP start and PEEP end, *P*-plateau Inspiratory plateau pressure, *Cpl* Respiratory system compliance, *Hyst* Tidal lung hysteresis, ΔHyst/ΔPEEP Change rate of tidal lung hysteresis, Dissipated E = dissipated energy per breath

### Tidal lung hysteresis and compliance at decremental PEEP levels: patterns of tidal recruitability

**Fig. 2:**
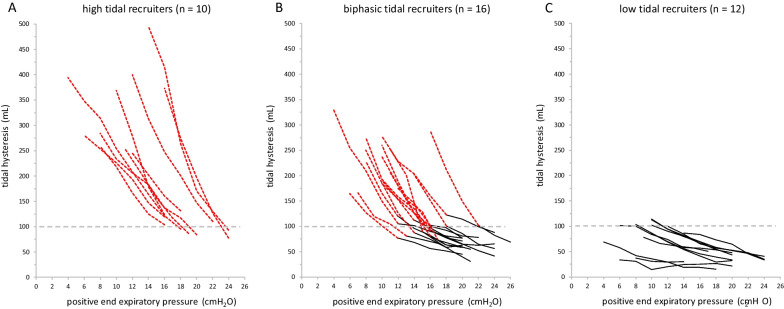
Tidal lung hysteresis during a decremental PEEP trial in high, biphasic and low tidal-recruiters. The large increase of tidal lung hysteresis between two PEEP levels is displayed as dotted red lines (panels A and B); a small increase of tidal lung hysteresis is displayed as continuous black lines (panels B and C). In consistently high and low tidal-recruiters the average ΔHyst/ΔPEEP was 25.9 ± 10.0 and 4.5 ± 1.6 (*p* < 0.0001) respectively. In the fast and in the slow phases that were observed in patients displaying a biphasic pattern, ΔHyst/ΔPEEP was 23.7 ± 7.0 and 5.6 ± 2.0 respectively. Lung hysteresis moved from 100.6 ± 20.0 to 335.4 ± 82.8 ml in high tidal-recruiters, from 35.7 ± 12.5 to 85.3 ± 27.0 ml in low tidal-recruiters and from 62.0 ± 14.9 to 230.5 ± 49.0 ml in biphasic pattern (*p* < 0.001). Lung hysteresis corresponding to the change in slope in the biphasic pattern was 95.6 ± 14.4 ml. Grey horizontal dotted lines mark the value of tidal hysteresis of 100 mL

According to tidal hysteresis, 10 (26%) patients were high tidal-recruiters, 12 (32%) patients were low tidal-recruiters and 16 (42%) patients displayed a biphasic pattern (Fig. 2). Low tidal-recruiters had similar clinical PEEP, driving pressure and plateau pressure compared to the other two patterns but they had longer hospital stay, longer mechanical ventilation and lower compliance (Table 1). Changes of respiratory mechanics observed during the decremental PEEP trial are detailed in Table 2. In high and low tidal-recruiters, ΔHyst/ΔPEEP was similar to the fast and the slow phase of biphasic pattern, respectively (Fig. 2). In the 208 PEEP steps-down that were analysed, compliance increased in 170 (82%) cases, and in 75/170 (44%) the increase of compliance was associated to a large increase of tidal lung hysteresis.

### PEEP values suggested by the best compliance vs. the combined approach

The best compliance and the combined approach did not agree on the direction of PEEP change (increase, keep constant or decrease) in 22 (58%) patients. Interrater agreement Cohen’s K between the two approaches was 0.024 (95% CI − 0.023–0.071). The best compliance approach suggested to increase, keep constant and decrease PEEP in 0, 1 and 37 patients. On average, PEEP_BC_ was 10.0 ± 3.2 cmH_2_O, with no differences among the three patterns (Table 3, Additional file 3: Fig. S3). The combined approach suggested to increase, keep constant and decrease PEEP in 13, 9 and 16 patients: the most frequent indication was to increase PEEP in high tidal-recruiters, to decrease PEEP in low tidal-recruiters and to keep PEEP constant in biphasic tidal-recruiters. On average, PEEP_CA_ was 14.6 ± 5.0 cmH_2_O (similar to clinical PEEP and higher than PEEP_BC_), being 19.5 ± 3.1, 8.9 ± 2.7 and 15.7 ± 2.5 cmH_2_O in high, low and biphasic patterns (*p* < 0.001). PEEP_BC_ and PEEP_CA_ were different in high and biphasic patterns, whereas they were identical in low tidal-recruiters (Table 3, Additional file 3: Fig. S3).

**Table 3:** Mechanical effects of different PEEP strategies in all patients and in subgroups with different mechanical patterns

	All patients *N* = 38 (100.0%)	High tidal-recruiters *N* = 10 (26.3%)	Biphasic tidal-recruiters *N* = 16 (42.1%)	Low tidal-recruiters *N* = 12 (31.6%)	*P* value
*Clinical setting*					
PEEP (cmH_2_O)	15.3 ± 2.5 ^§^	16.3 ± 2.8 ^# §^	15.3 ± 2.5 ^§^	14.4 ± 2.2 ^# §^	0.220
Cpl (mL/cmH_2_O)	37.1 ± 13.7 ^§^	50.3 ± 11.2 ^# §^	39.8 ± 7.9 ^§^	22.5 ± 6.6 ^# §^	< 0.001
Hyst (mL)	105.1 ± 50.2 ^§^	162.5 ± 32.7 ^# §^	105.4 ± 33.6 ^§^	57.0 ± 22.8 ^# §^	< 0.001
Dissipated E (J)	0.2 ± 0.1 ^§^	0.2 ± 0.1 ^# §^	0.2 ± 0.1 ^§^	0.1 ± 0.0 ^# §^	< 0.001
*Best Cpl approach*					
PEEP (cmH_2_O)	10.0 ± 3.2 ^ ^§^	11.5 ± 3.5 ^ ^§^	9.8 ± 3.0 ^ ^§^	8.9 ± 2.7 ^§^	0.156
PEEP change * (n)	0/1/37 ^	0/1/9 ^	0/0/16 ^	0/0/12	0.946
Cpl (mL/cmH_2_O)	44.8 ± 17.2 ^ ^§^	58.7 ± 16.0 ^ ^§^	49.1 ± 11.7 ^ ^§^	27.5 ± 8.5 ^§^	< 0.001
Hyst (mL)	204.7 ± 110.0 ^ ^§^	312.0 ± 110.2 ^ ^§^	227.3 ± 49.2 ^ ^§^	85.3 ± 27.0 ^§^	< 0.001
Dissipated E (J)	0.4 ± 0.2 ^ ^§^	0.6 ± 0.2 ^ ^§^	0.5 ± 0.1 ^ ^§^	0.1 ± 0.1 ^§^	< 0.001
*Combined approach*					
PEEP (cmH_2_O)	14.6 ± 5.0 ^	19.5 ± 3.1 ^ ^#^	15.7 ± 2.5 ^	8.9 ± 2.7 ^#^	< 0.001
PEEP change * (*n*)	13/9/16 ^	8/2/0 ^	5/7/4 ^	0/0/12	< 0.001
Cpl (ml/cmH_2_O)	36.4 ± 10.1 ^	42.8 ± 7.3 ^ ^#^	39.1 ± 8.2 ^	27.5 ± 8.5 ^#^	< 0.001
Hyst (mL)	92.7 ± 20.9 ^	100.6 ± 20.0 ^ ^#^	93.2 ± 14.8 ^	85.3 ± 27.0 ^#^	0.235
Dissipated E (J)	0.1 ± 0.1 ^	0.1 ± 0.0 ^ ^#^	0.1 ± 0.0 ^	0.1 ± 0.1 ^#^	0.268

*PEEP change results are expressed as the absolute number of patients in whom it was suggested to increase/keep constant/decrease PEEP by the best compliance or the combined approach. *PEEP* Positive end-expiratory pressure, *Cpl* Respiratory system compliance, *Hyst* Tidal lung hysteresis, *Dissipated E* Dissipated energy per breath. *P* values in the table are related to the comparison among the three patterns. ^ *p* < 0.01 best compliance approach vs. combined approach; ^#^ *p* < 0.01 combined approach vs. clinical PEEP; ^§^ *p* < 0.01 best compliance approach vs. clinical PEEP

### Mechanical effects of different PEEP strategies

The effects on respiratory mechanics of clinical PEEP, PEEP_BC_ and PEEP_CA_ are detailed in Table 3 and in Additional file 4: Figs. S4 and Additional file 5: Fig. S5. Clinical PEEP was associated with a tidal lung hysteresis of 105.1 ± 50.2 mL: high tidal-recruiters showed the highest values and low tidal-recruiters showed the lowest ones (*p* < 0.001). At clinical PEEP, dissipated energy was 0.2 ± 0.1 J with low tidal recruiters showing values lower than high and biphasic tidal recruiters (*p* < 0.001). Hyst and dissipated energy associated with PEEP_CA_ were 92.7 ± 20.9 mL and 0.1 ± 0.1 J respectively, with no differences among the three patterns. Hyst and dissipated energy associated with PEEP_BC_ were 204.7 ± 110.0 mL and 0.4 ± 0.2 J respectively, higher than the values observed with clinical PEEP and PEEP_CA_ (*p* < 0.001).

### Tidal lung hysteresis to predict tidal recruitment after a PEEP change

Absolute values of tidal lung hysteresis were higher in high than in low tidal-recruiters: along the decremental PEEP trial, Hyst increased from 100.6 ± 20.0 to 335.4 ± 82.8 mL in high tidal-recruiters and from 35.7 ± 12.5 to 85.3 ± 27.0 mL in low tidal-recruiters (*p* < 0.001). Tidal lung hysteresis corresponding to the change in slope in the biphasic pattern was 95.6 ± 14.4 ml. Data distribution suggested that a threshold value of tidal hysteresis of 100 mL could be used to separate high from low tidal-recruiters, as well as the fast from the slow phase in biphasic pattern (Additional file 6: Fig. S6). Area under the curve for the absolute value of tidal hysteresis as a predictor of large increase of Hyst after the PEEP change was 0.97 (*p* < 0.001): tidal lung hysteresis ≥ 100 mL had 83.0% sensitivity and 96.5% specificity in predicting a large increase of tidal recruitment at the next PEEP step-down (Additional file 7: Fig. S7).

## Discussion

The conventional pressure–volume loop, obtained with a single manoeuver from zero to high values of airway pressure, provides information on lung recruitability that physicians can use to choose a “high” versus “low” PEEP strategy [14, 15]; however, this information alone is not useful for fine PEEP titration. On the other hand, the conventional decremental PEEP trial may be used to personalize PEEP in ARDS patients, but its interpretation can be complicated by tidal recruitment [5, 8–10]. As a solution, we designed a new approach combining the two techniques, where a pressure–volume loop between PEEP and plateau pressure was obtained at each step of a decremental PEEP trial. In this new approach, lung hysteresis was used to detect tidal recruitment and to allow a correct interpretation of changes of compliance when PEEP was step-by–step reduced during the trial.

The main findings of the present study were: (1) according to their propensity to tidal lung recruitability, patients could be grouped into three patterns: high tidal-recruiters, low tidal-recruiters and biphasic pattern; (2) after a PEEP decrease, an improvement of compliance was frequently associated with large increase of tidal lung hysteresis suggesting tidal recruitment; (3) a combined approach substantially modified the interpretation of the PEEP trial in both high and biphasic tidal-recruiters, with the potential to limit the energy dissipated into the lung tissue at each mechanical breath (4) to predict the risk of tidal recruitment associated to a PEEP reduction, a threshold value of tidal lung hysteresis was found.

### Tidal lung hysteresis at decremental PEEP levels

According to tidal lung hysteresis, 2/3 of our patients showed substantial tidal recruitment when PEEP was reduced. This finding could have been promoted by two facts. First, in all our patients ARDS was related to COVID-19: high lung recruitability has often been observed in this condition [23, 24]. Second, the decremental PEEP trial as here performed – with constant plateau pressure and incremental driving pressure – magnifies tidal recruitability as compared to a decremental PEEP trial with constant driving pressure and decremental plateau pressure. In fact, in this latter way the applied “opening” pressure decreases at each PEEP step down: this is supposed to decrease the amount of lung tissue that is recruited at the end of inspiration as well as the corresponding level of PEEP that is needed to keep this lung tissue open during expiration [13, 21]. Our findings are consistent with previous experimental observations. In a model of acute lung injury, tidal lung hysteresis largely and progressively increased in washed lungs that were inflated to the same plateau pressure (30 cmH_2_O) after being deflated to decreasing levels of PEEP, thus applying increasing values of driving pressure [20]. The changes of lung hysteresis when an increasing driving pressure is applied were also studied in an isolated lung animal model [13]. In these experiments, high and low intra-tidal recruitability were obtained by applying a negative vs. a positive end-expiratory transpulmonary pressure (P_L_) in normal lungs. When the driving pressure was progressively increased, tidal lung hysteresis showed a small increase in case of positive end-expiratory P_L_ (low tidal recruitability) and a large increase in case of negative end-expiratory P_L_ (high tidal recruitability).

### Changes of compliance at decremental PEEP levels

Improvements in compliance were frequently associated to large increases in tidal hysteresis in our patients. This is consistent with previous findings of a higher compliance at low PEEP vs. high PEEP not only in low recruiters but also in high recruiters [6]: in these latter patients, tidal recruitment is the likely explanation. Moreover, “normal” compliance associated to extensive lung involvement was frequently observed in COVID-19 patients [25]. Accordingly, these patients showed higher compliance despite similar lung weight compared to patients with non-COVID-19 ARDS [26]. Our findings confirm that the information provided by the compliance can be misleading in ARDS patients. A PEEP setting unable to prevent expiratory derecruitment and cyclic opening-closure of distal airways and alveoli results in increased estimate of compliance. Thus, when an apparently good compliance is observed, particularly if associated with an extensive lung involvement and a severely compromised oxygenation, large tidal recruitment should be ruled out before embracing a low PEEP strategy [27].

### Interpretation of a decremental PEEP trial: best compliance vs. combined approach

Both clinical PEEP and PEEP_BC_ were similar in patients with high vs. low tidal recruitability, whereas the combined approach promoted a personalized PEEP setting according to the specific risk of tidal recruitment of each patient. In fact, PEEP_CA_ can be interpreted as the lowest PEEP value able to avoid tidal recruitment and limit tidal lung hysteresis. From a physics perspective tidal lung hysteresis corresponds to dissipated energy, that is the amount of energy transferred to the respiratory system during inflation that is not recovered during deflation [28]. Therefore, the combined approach has the potential to limit the energy that is dissipated into the lung tissue at each mechanical breath in patients at risk of tidal recruitment. As predictable, PEEP_BC_ and PEEP_CA_ were identical in patients with low tidal recruitability: when the confounding effect of tidal recruitment is ruled out, a better compliance can be safely interpreted as a favourable mechanical effect of a PEEP decrease. Mechanical power was recently described to assess the overall intensity of mechanical ventilation [29]. However, it is debated whether to include the elastic component related to PEEP in the computation of mechanical power [30]. In fact, an increase of PEEP always translates into an increase of the total energy transferred to the respiratory system during mechanical inflation with constant driving pressure. On the other hand, a higher PEEP can prevent tidal recruitment and limit the amount of dissipated energy. The net mechanical effect will be in favour of a higher PEEP if its positive effect (lower dissipated energy) overcomes the negative one (higher total energy). Tidal lung hysteresis may help evaluate PEEP at the bedside by providing an objective measure of the –until now- unknown variable i.e., the amount of dissipated energy.

### Threshold value of tidal lung hysteresis

In our patients, a value of tidal hysteresis below 100 mL was almost invariably associated to low tidal recruitability, suggesting that the tested PEEP level was effective in preventing expiratory collapse and cyclic opening-closing of alveoli. Tidal lung hysteresis could eventually be measured outside of a decremental PEEP trial, to get information on current clinical settings of mechanical ventilation. This opportunity is particularly attractive, being the procedure fast (10–15 s) and inherently safe, because the manoeuver is designed to investigate the same range of alveolar pressures of tidal ventilation. Whether the threshold found in our study could apply also to other settings, has to be verified.

### Study limitations

We studied a population of COVID-19 ARDS patients at different timing after intubation: our findings should be confirmed in other settings. The decremental PEEP trial adopted in the present study increased the propensity to tidal recruitment and therefore its confounding effect on compliance: with a different technique, agreement between the best compliance and the combined approach could also be different. Moreover, in some patient (mainly low tidal recruiter) the value of PEEP selected by the combined approach was tested -during the trial- in association with a driving pressure that was larger than the one applied after the trial: some overestimation of the lowest PEEP value still able to avoid tidal recruitment was possible in this case. We did not systematically measure esophageal pressure, therefore we cannot provide insights about the relationship between transpulmonary pressure and tidal recruitment in our patients. We focused on mechanical effects of PEEP: the impact on gas exchanges and hemodynamics of different PEEP values was not investigated.

## Conclusions

The assessment of tidal lung hysteresis provides information potentially useful to personalize mechanical ventilation in ARDS patients, with the aim of limiting tidal recruitment and the energy dissipated into the respiratory system at each mechanical breath. A combined approach, based on compliance and tidal hysteresis, may help select the PEEP value associated with the best compromise among lung hyperinflation, lung derecruitment and atelectrauma. Whether this will lead to any clinical benefit has yet to be investigated.

## Supplementary Information

**Additional file 1. Fig S1. Schematic representation of a decremental PEEP trial with low-flow inflation-deflation manoeuvres at each PEEP level.** After a recruitment manoeuvre, PEEP was set at the clinical PEEP + 6 cmH_2_O (PEEP start) and decreased by 2 cmH_2_O every 3 min until clinical PEEP – 6 cmH_2_O (PEEP end)). The range of PEEP tested could be set differently by the attending physician (lower PEEP start and/or higher PEEP end) in case of hemodynamic instability and/or substantial desaturation. The driving pressure (ΔP) was set at PEEP start to keep plateau pressure close to 30 cmH2O. Plateau pressure was kept constant throughout the decremental PEEP trial, therefore the applied ΔP increased by 2 cmH_2_O at each step. Respiratory rate was adjusted along the trial in order to have at end inspiration and at end expiration a near zero-flow condition (i.e., an alveolar pressure reflected by airway pressure) and to keep end-tidal CO_2_ reasonably constant. At the end of each PEEP step, a low-flow inflation/deflation manoeuvre was performed, from the PEEP to plateau pressure and back.

**Additional file 2. Fig S2. Rate of change of tidal hysteresis in high, low and biphasic tidal-recruiters.** Box and whisker plot showing median value, interquartile range, upper and lower extreme values of rate of change of tidal hysteresis; outliers are displayed as open circles. High = high tidal-recruiters showing consistently large increase of tidal hysteresis at each PEEP step-down; Low = low tidal-recruiters showing consistently small increase of tidal hysteresis at each PEEP step-down; Biph_F = fast phase of patients with biphasic pattern; Biph_S = slow phase of patients with biphasic pattern. The grey dotted line marks the value of ΔHysteresis / ΔPEEP of 10 mL / cmH_2_O.

**Additional file 3. Fig S3. PEEP values: clinical setting, best compliance approach and combined approach.** Data are provided for all patients and for the three patterns of tidal recruitability. The combined approach suggested different PEEP values in patients with different propensity to tidal recruitment (*p* < 0.001), whereas clinical PEEP and the PEEP value suggested by the best compliance approach did not differ among the three patterns of tidal recruitability. Compared to clinical PEEP, the combined approach suggested a higher PEEP in high tidal recruiters (*p* < 0.01), a lower PEEP in low tidal recruiters (*p* < 0.01) and similar PEEP in biphasic pattern: overall, clinical PEEP and PEEP with the combined approach did not differ. Compared to the best compliance approach, PEEP suggested by the combined approach was higher in high tidal recruiters (*p* < 0.01) and in biphasic pattern (*p* < 0.01) and identical in low tidal recruiters.

**Additional file 4. Fig S4. Respiratory system compliance with different PEEP settings: clinical setting, best compliance approach and combined approach.** Data are provided for all patients and for the three patterns of tidal recruitability. Compliance was different in patients with different propensity to tidal recruitment (*p* < 0.001) whatever the approach to set PEEP (clinical, best compliance, or combined approach). Compared to clinical PEEP, compliance with the combined approach was lower in high tidal recruiters (*p* < 0.01), higher in low tidal recruiters (*p* < 0.01) and similar in biphasic pattern: overall, compliance did not differ with clinical PEEP vs. PEEP suggested by the combined approach. Compared to the best compliance approach, compliance with the combined approach was lower in high tidal recruiters (*p* < 0.01) and in biphasic pattern (*p* < 0.01) and identical in low tidal recruiters.

**Additional file 5. Fig S5. Tidal lung hysteresis with different PEEP settings: clinical setting, best compliance approach and combined approach.** Data are provided for all patients and for the three patterns of tidal recruitability. Tidal hysteresis was different in patients with different propensity to tidal recruitment with clinical PEEP (*p* < 0.001) and with PEEP suggested by the best compliance approach (*p* < 0.001); when PEEP was set according to the combined approach, tidal hysteresis did not differ among the three patterns of tidal recruitability, being close to 100 mL in all cases. Compared to clinical PEEP, with the combined approach tidal hysteresis was lower in high tidal recruiters (*p* < 0.01), higher in low tidal recruiters (*p* < 0.01) and similar in biphasic pattern. Compared to the best compliance approach, tidal hysteresis with the combined approach was lower in high tidal recruiters (*p* < 0.01) and in biphasic pattern (*p* < 0.01) and identical in low tidal recruiters.

**Additional file 6. Fig S6. Tidal hysteresis in high, low and biphasic tidal-recruiters.** Box and whisker plot showing median value, interquartile range, upper and lower extreme values of tidal hysteresis; outliers are displayed as open circles. High = high tidal-recruiters showing consistently large increase of tidal hysteresis at each PEEP step-down; Low = low tidal-recruiters showing consistently small increase of tidal hysteresis at each PEEP step-down; Biph_F = fast phase of patients with biphasic pattern; Biph_S = slow phase of patients with biphasic pattern. The grey dotted line marks the value of Tidal Hysteresis of 100 mL.

**Additional file 7. Fig S7. Tidal lung hysteresis to predict tidal recruitment after PEEP step-down: ROC curve.** Area under the curve for the absolute value of tidal lung hysteresis as a predictor of a large increase of tidal lung hysteresis after a change of PEEP was 0.97 (*p* < 0.001). Tidal lung hysteresis ≥ 100 ml had 83.0% sensitivity and 96.5% specificity in predicting a large increase of tidal recruitment at next PEEP step-down.

## Data Availability

By e-mail to the corresponding author.
