# Supplementary material for: Tidal lung hysteresis to interpret PEEP-induced changes in compliance in ARDS patients
Source: Crit Care. 2023 Jun 13;27:233. doi: 10.1186/s13054-023-04506-6 (PMC10261834; doi:10.1186/s13054-023-04506-6)
Supplement: Supplementary file 1 — Additional file 1. Fig S1. Schematic representation of a decremental PEEP trial with low-flow inflation-deflation manoeuvres at each PEEP level. After a recruitment manoeuvre, PEEP was set at the clinical PEEP + 6 cmH2O (PEEP start) and decreased by 2 cmH2O every 3 min until clinical PEEP – 6 cmH2O (PEEP end)). The range of PEEP tested could be set differently by the attending physician (lower PEEP start and/or higher PEEP end) in case of hemodynamic instability and/or substantial desaturation. The driving pressure (ΔP) was set at PEEP start to keep plateau pressure close to 30 cmH2O. Plateau pressure was kept constant throughout the decremental PEEP trial, therefore the applied ΔP increased by 2 cmH2O at each step. Respiratory rate was adjusted along the trial in order to have at end inspiration and at end expiration a near zero-flow condition (i.e., an alveolar pressure reflected by airway pressure) and to keep end-tidal CO2 reasonably constant. At the end of each PEEP step, a low-flow inflation/deflation manoeuvre was performed, from the PEEP to plateau pressure and back. [file 13054_2023_4506_MOESM1_ESM.pptx]

## Slide 1
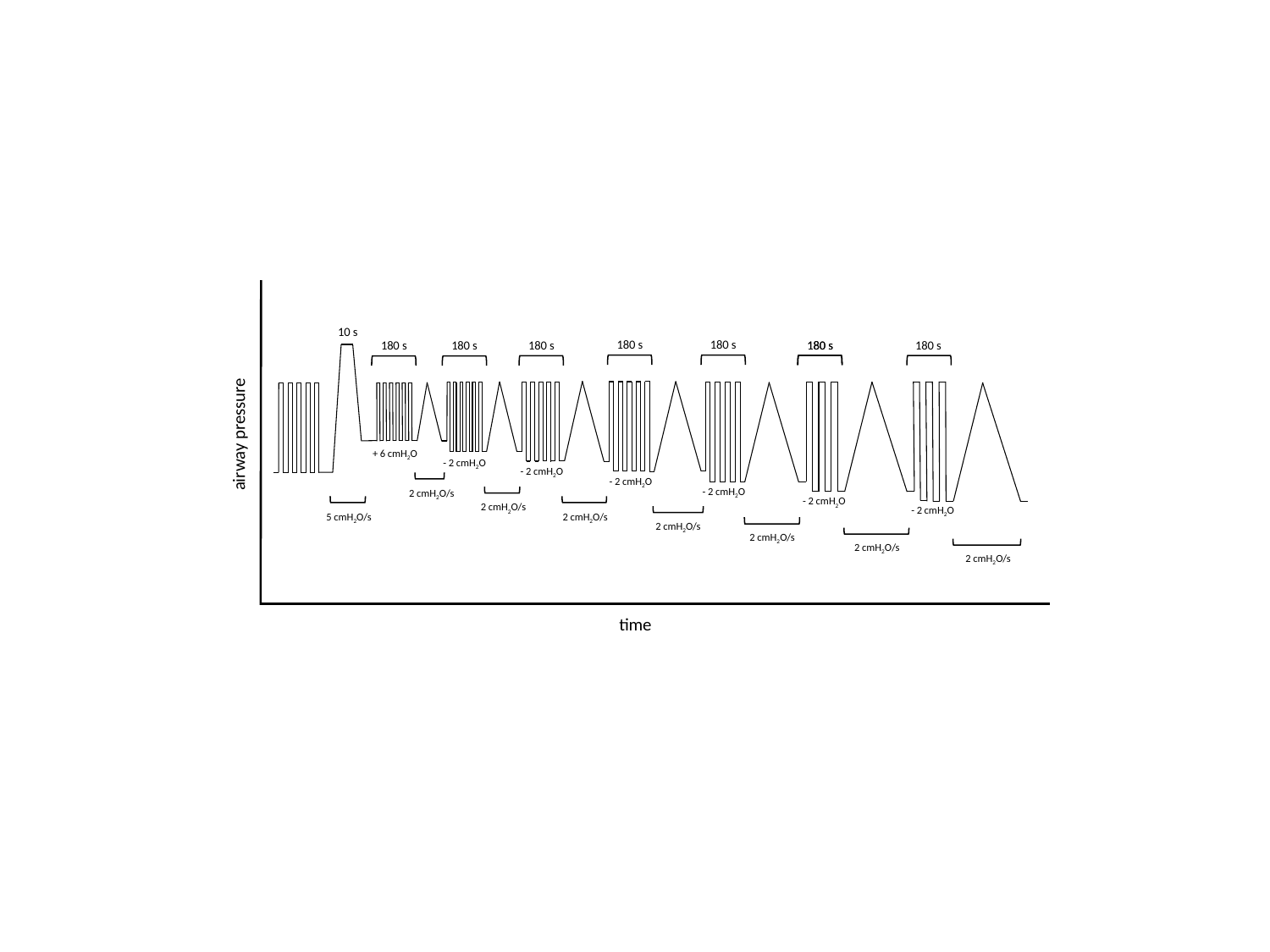

10 s
180 s
180 s
180 s
180 s
180 s
180 s
180 s
180 s
airway pressure
+ 6 cmH2O
- 2 cmH2O
- 2 cmH2O
- 2 cmH2O
- 2 cmH2O
2 cmH2O/s
- 2 cmH2O
2 cmH2O/s
- 2 cmH2O
5 cmH2O/s
2 cmH2O/s
2 cmH2O/s
2 cmH2O/s
2 cmH2O/s
2 cmH2O/s
time
