# Supplementary figures and images for: Tidal lung hysteresis to interpret PEEP-induced changes in compliance in ARDS patients
Source: Crit Care. 2023 Jun 13;27:233. doi: 10.1186/s13054-023-04506-6 (PMC10261834; doi:10.1186/s13054-023-04506-6)

## Slide 1
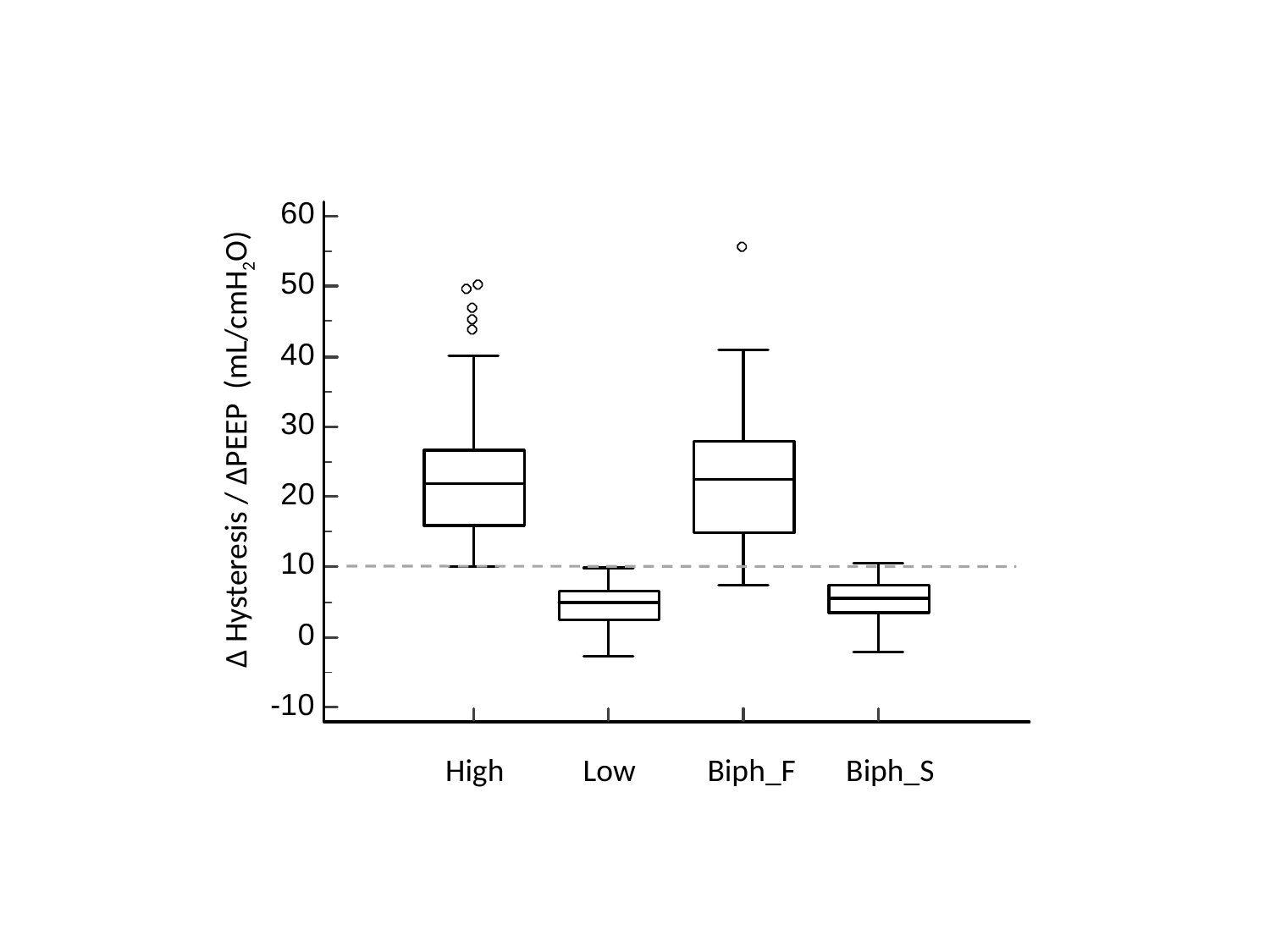

Δ Hysteresis / ΔPEEP (mL/cmH2O)
High Low Biph_F Biph_S

Supplement: Supplementary file 2 — Additional file 2. Fig S2. Rate of change of tidal hysteresis in high, low and biphasic tidal-recruiters. Box and whisker plot showing median value, interquartile range, upper and lower extreme values of rate of change of tidal hysteresis; outliers are displayed as open circles. High = high tidal-recruiters showing consistently large increase of tidal hysteresis at each PEEP step-down; Low = low tidal-recruiters showing consistently small increase of tidal hysteresis at each PEEP step-down; Biph_F = fast phase of patients with biphasic pattern; Biph_S = slow phase of patients with biphasic pattern. The grey dotted line marks the value of ΔHysteresis / ΔPEEP of 10 mL / cmH2O. [file 13054_2023_4506_MOESM2_ESM.pptx]

## Slide 1
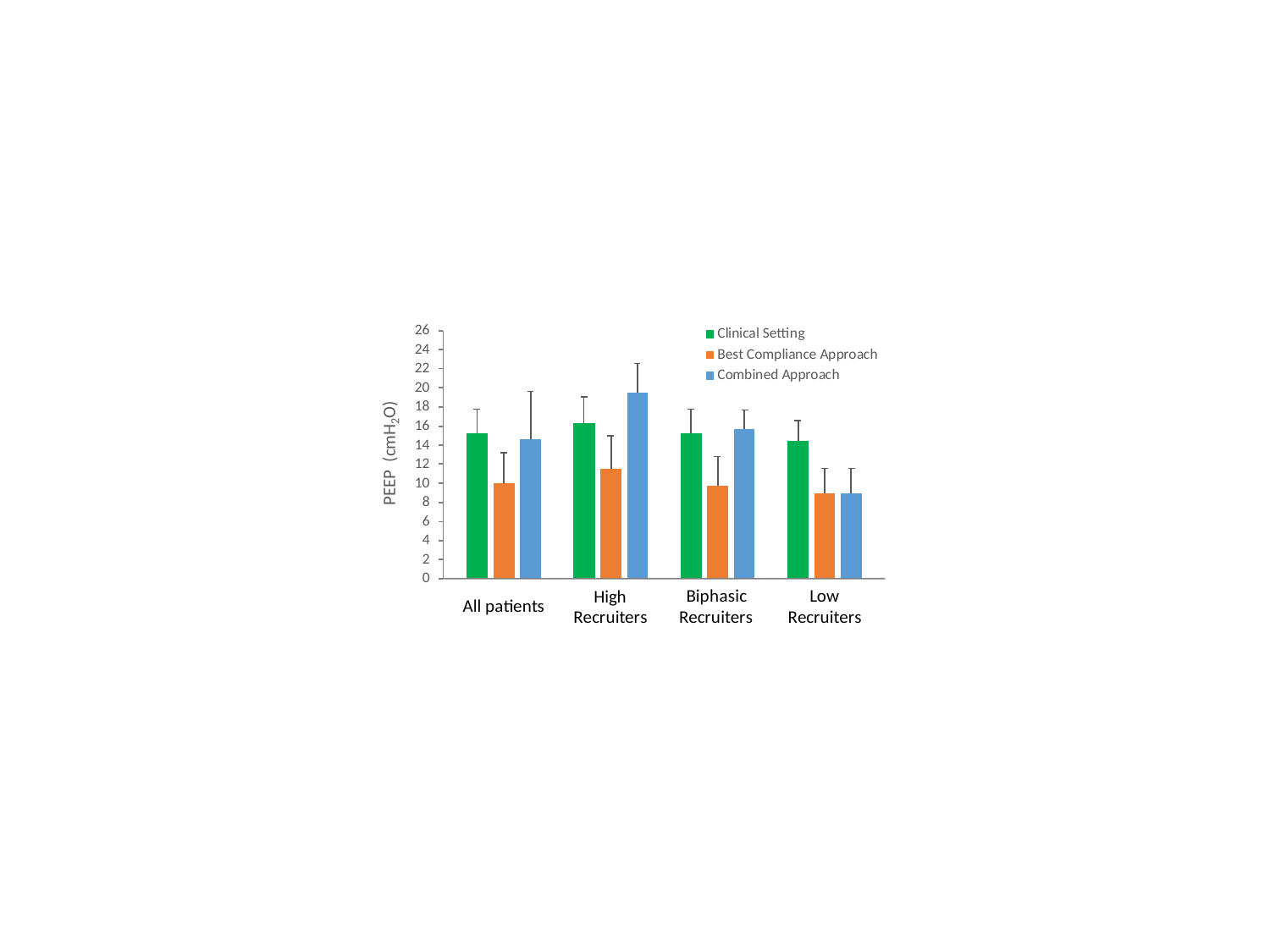

Supplement: Supplementary file 3 — Additional file 3. Fig S3. PEEP values: clinical setting, best compliance approach and combined approach. Data are provided for all patients and for the three patterns of tidal recruitability. The combined approach suggested different PEEP values in patients with different propensity to tidal recruitment (p < 0.001), whereas clinical PEEP and the PEEP value suggested by the best compliance approach did not differ among the three patterns of tidal recruitability. Compared to clinical PEEP, the combined approach suggested a higher PEEP in high tidal recruiters (p < 0.01), a lower PEEP in low tidal recruiters (p < 0.01) and similar PEEP in biphasic pattern: overall, clinical PEEP and PEEP with the combined approach did not differ. Compared to the best compliance approach, PEEP suggested by the combined approach was higher in high tidal recruiters (p < 0.01) and in biphasic pattern (p < 0.01) and identical in low tidal recruiters. [file 13054_2023_4506_MOESM3_ESM.pptx]

## Slide 1
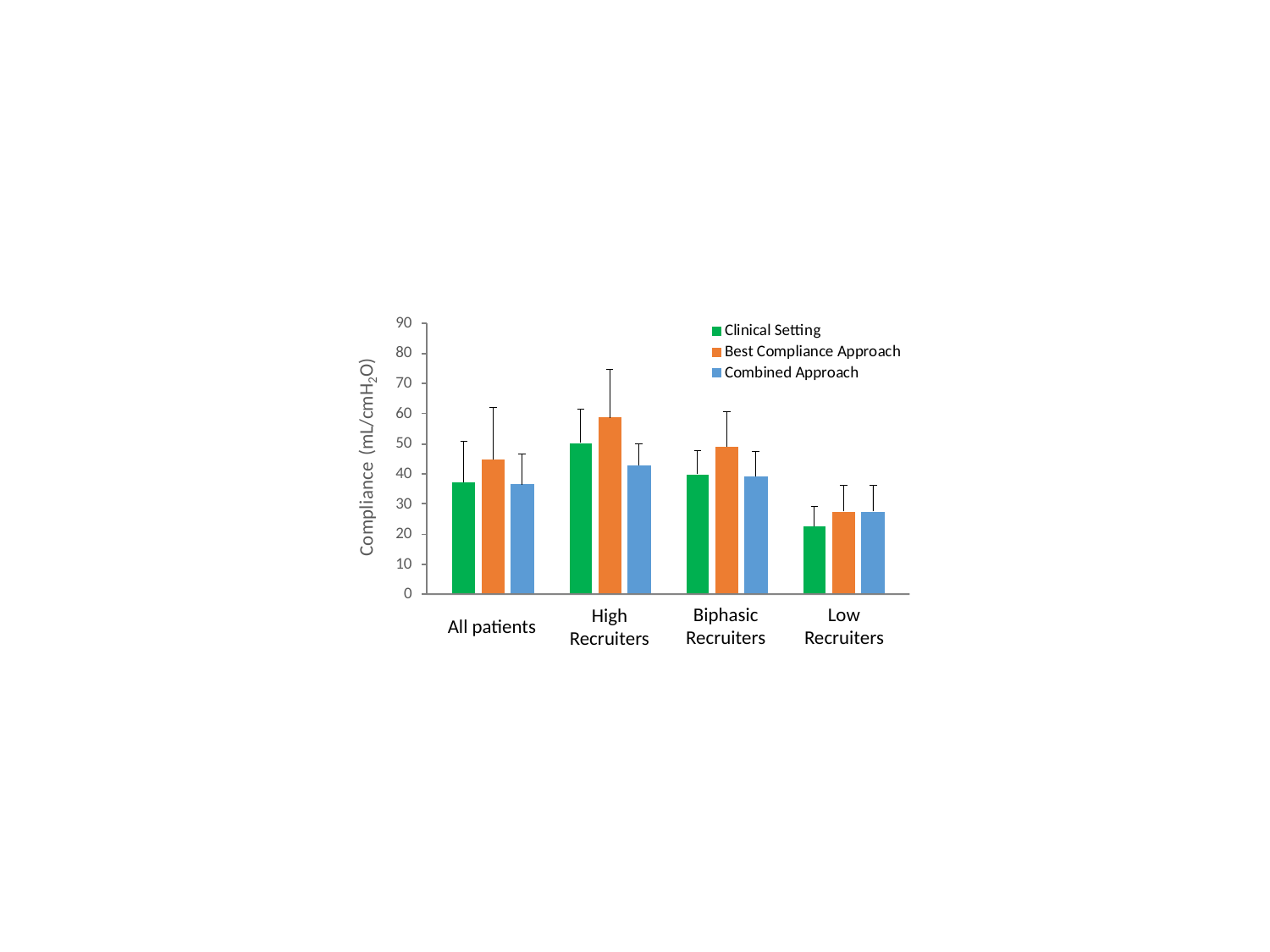

Biphasic Recruiters
Low Recruiters
High Recruiters
All patients

Supplement: Supplementary file 4 — Additional file 4. Fig S4. Respiratory system compliance with different PEEP settings: clinical setting, best compliance approach and combined approach. Data are provided for all patients and for the three patterns of tidal recruitability. Compliance was different in patients with different propensity to tidal recruitment (p < 0.001) whatever the approach to set PEEP (clinical, best compliance, or combined approach). Compared to clinical PEEP, compliance with the combined approach was lower in high tidal recruiters (p < 0.01), higher in low tidal recruiters (p < 0.01) and similar in biphasic pattern: overall, compliance did not differ with clinical PEEP vs. PEEP suggested by the combined approach. Compared to the best compliance approach, compliance with the combined approach was lower in high tidal recruiters (p < 0.01) and in biphasic pattern (p < 0.01) and identical in low tidal recruiters. [file 13054_2023_4506_MOESM4_ESM.pptx]

## Slide 1
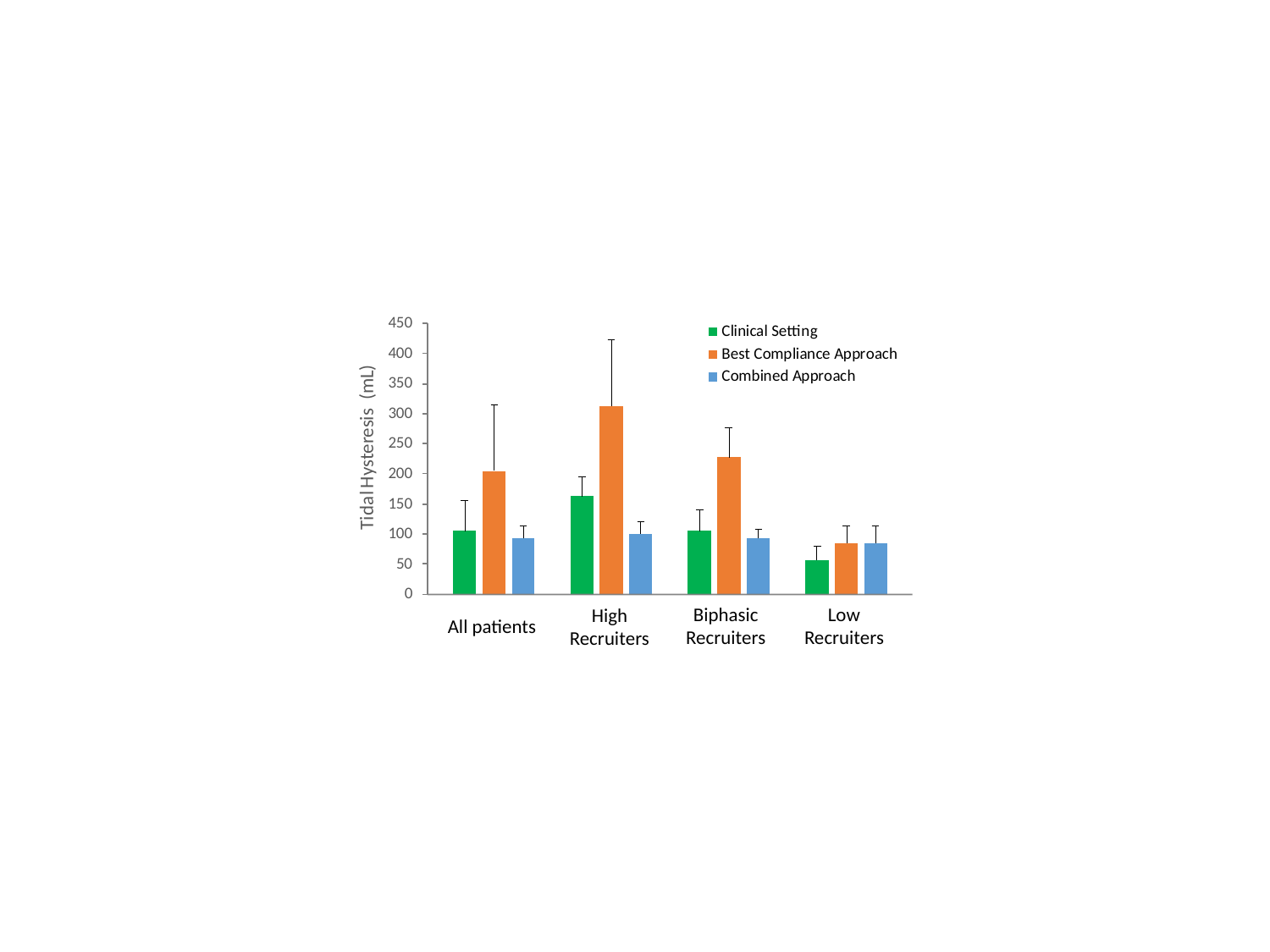

Biphasic Recruiters
Low Recruiters
High Recruiters
All patients

Supplement: Supplementary file 5 — Additional file 5. Fig S5. Tidal lung hysteresis with different PEEP settings: clinical setting, best compliance approach and combined approach. Data are provided for all patients and for the three patterns of tidal recruitability. Tidal hysteresis was different in patients with different propensity to tidal recruitment with clinical PEEP (p < 0.001) and with PEEP suggested by the best compliance approach (p < 0.001); when PEEP was set according to the combined approach, tidal hysteresis did not differ among the three patterns of tidal recruitability, being close to 100 mL in all cases. Compared to clinical PEEP, with the combined approach tidal hysteresis was lower in high tidal recruiters (p < 0.01), higher in low tidal recruiters (p < 0.01) and similar in biphasic pattern. Compared to the best compliance approach, tidal hysteresis with the combined approach was lower in high tidal recruiters (p < 0.01) and in biphasic pattern (p < 0.01) and identical in low tidal recruiters. [file 13054_2023_4506_MOESM5_ESM.pptx]

## Slide 1
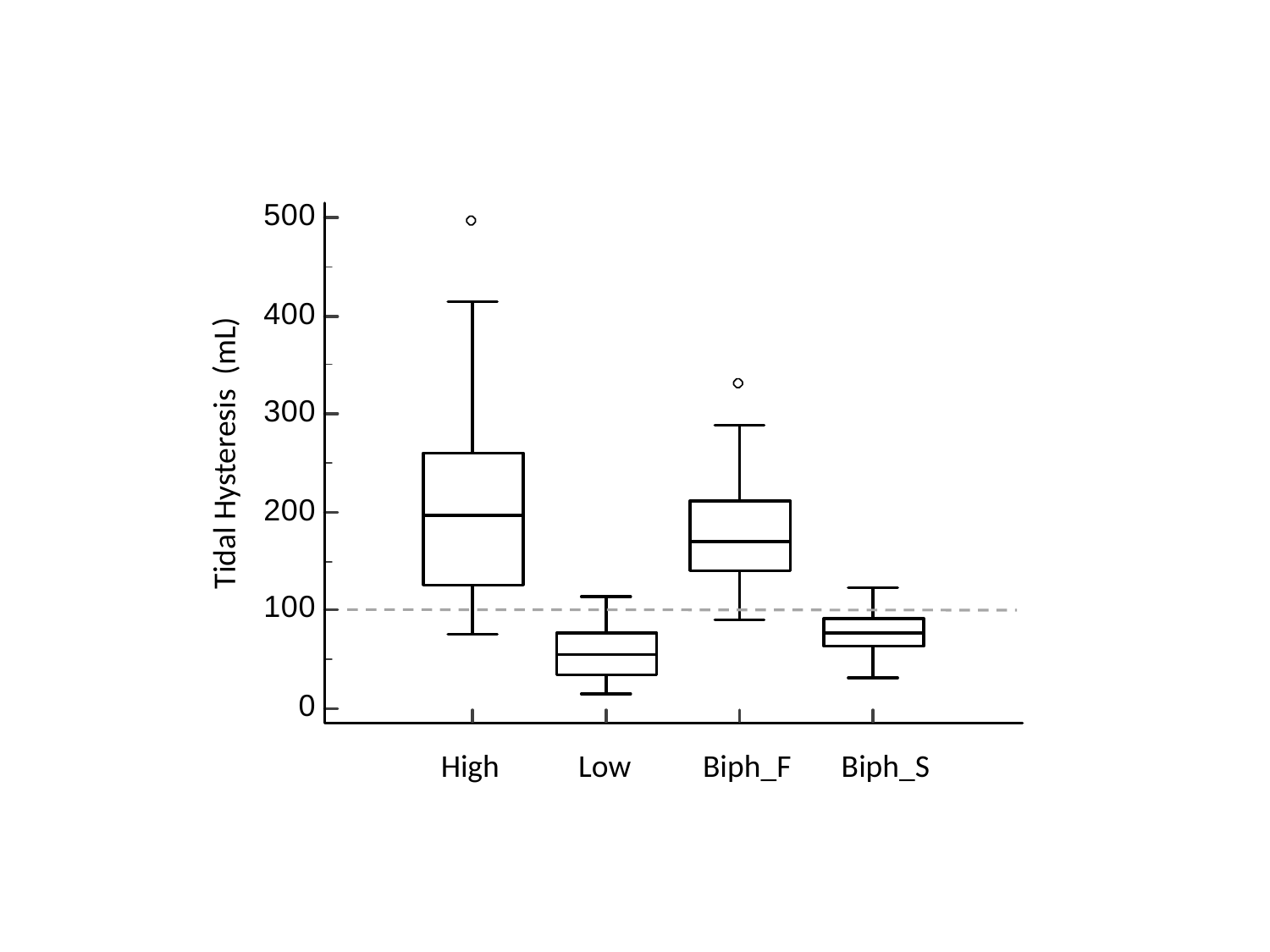

Tidal Hysteresis (mL)
High Low Biph_F Biph_S

Supplement: Supplementary file 6 — Additional file 6. Fig S6. Tidal hysteresis in high, low and biphasic tidal-recruiters. Box and whisker plot showing median value, interquartile range, upper and lower extreme values of tidal hysteresis; outliers are displayed as open circles. High = high tidal-recruiters showing consistently large increase of tidal hysteresis at each PEEP step-down; Low = low tidal-recruiters showing consistently small increase of tidal hysteresis at each PEEP step-down; Biph_F = fast phase of patients with biphasic pattern; Biph_S = slow phase of patients with biphasic pattern. The grey dotted line marks the value of Tidal Hysteresis of 100 mL. [file 13054_2023_4506_MOESM6_ESM.pptx]

## Slide 1
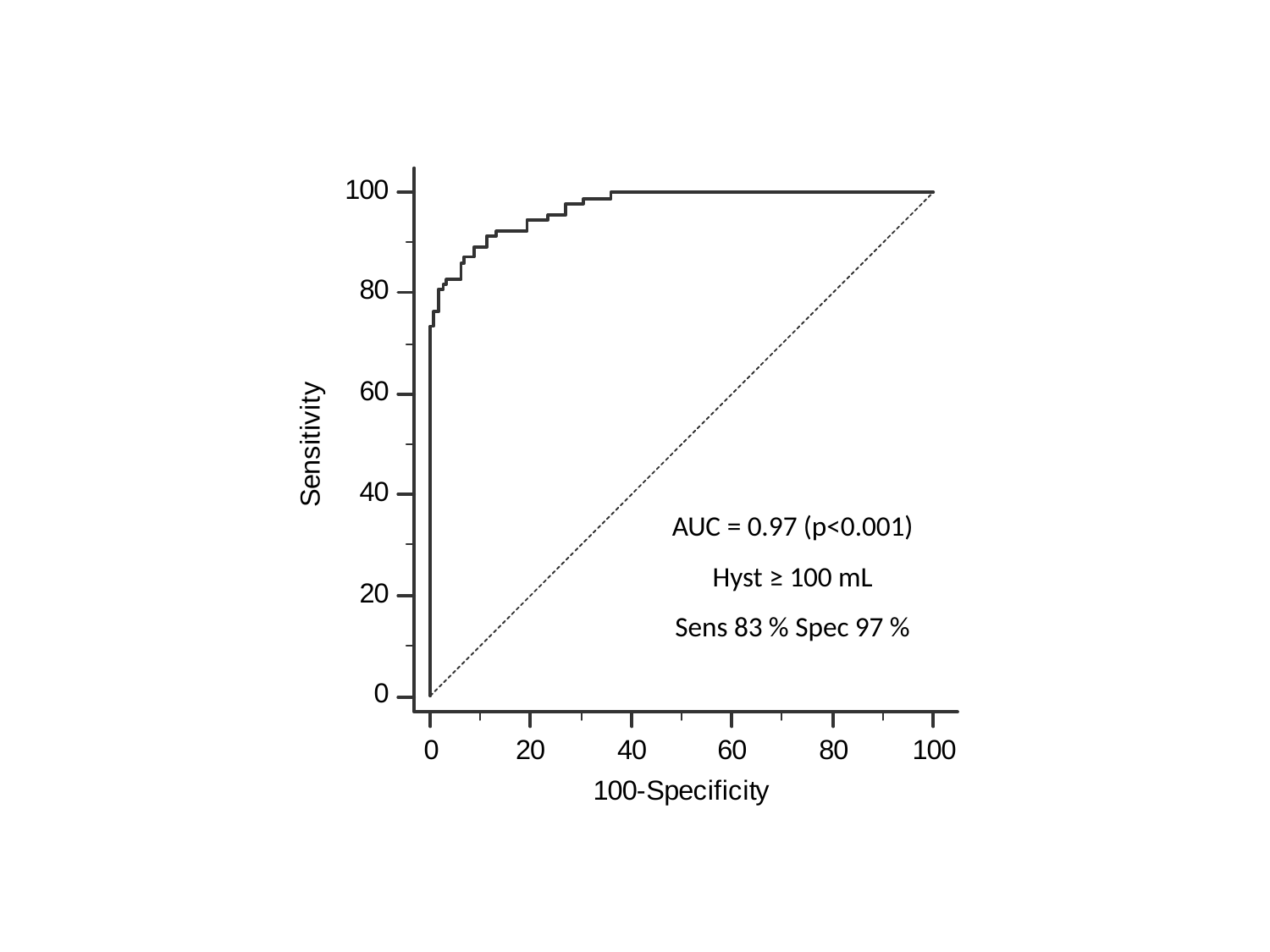

AUC = 0.97 (p<0.001)
Hyst ≥ 100 mL
Sens 83 % Spec 97 %

Supplement: Supplementary file 7 — Additional file 7. Fig S7. Tidal lung hysteresis to predict tidal recruitment after PEEP step-down: ROC curve. Area under the curve for the absolute value of tidal lung hysteresis as a predictor of a large increase of tidal lung hysteresis after a change of PEEP was 0.97 (p < 0.001). Tidal lung hysteresis ≥ 100 ml had 83.0% sensitivity and 96.5% specificity in predicting a large increase of tidal recruitment at next PEEP step-down. [file 13054_2023_4506_MOESM7_ESM.pptx]
